# Longitudinal Changes in Depressive Circuitry in Response to Neuromodulation Therapy

**DOI:** 10.3389/fncir.2016.00050

**Published:** 2016-07-29

**Authors:** Yagna Pathak, Oludamilola Salami, Sylvain Baillet, Zhimin Li, Christopher R. Butson

**Affiliations:** ^1^Department of Biomedical Engineering, Marquette UniversityMilwaukee, WI, USA; ^2^Department of Psychiatry, Medical College of WisconsinMilwaukee, WI, USA; ^3^McConnell Brain Imaging Centre, Montreal Neurological Institute, McGill UniversityMontreal, QC, Canada; ^4^Department of Neurology, Medical College of WisconsinMilwaukee, WI, USA; ^5^Department of Bioengineering, Scientific Computing and Imaging (SCI) Institute, University of UtahSalt Lake City, UT, USA

**Keywords:** repetitive transcranial magnetic stimulation (rTMS), neuromodulation, dorsolateral prefrontal cortex (DLPFC), depression, deep brain stimulation (DBS), functional connectivity

## Abstract

**Background**: Major depressive disorder (MDD) is a public health problem worldwide. There is increasing interest in using non-invasive therapies such as repetitive transcranial magnetic stimulation (rTMS) to treat MDD. However, the changes induced by rTMS on neural circuits remain poorly characterized. The present study aims to test whether the brain regions previously targeted by deep brain stimulation (DBS) in the treatment of MDD respond to rTMS, and whether functional connectivity (FC) measures can predict clinical response.

**Methods**: rTMS (20 sessions) was administered to five MDD patients at the left-dorsolateral prefrontal cortex (L-DLPFC) over 4 weeks. Magnetoencephalography (MEG) recordings and Montgomery-Asberg depression rating scale (MADRS) assessments were acquired before, during and after treatment. Our primary measures, obtained with MEG source imaging, were changes in power spectral density (PSD) and changes in FC as measured using coherence.

**Results**: Of the five patients, four met the clinical response criterion (40% or greater decrease in MADRS) after 4 weeks of treatment. An increase in gamma power at the L-DLPFC was correlated with improvement in symptoms. We also found that increases in delta band connectivity between L-DLPFC/amygdala and L-DLPFC/pregenual anterior cingulate cortex (pACC), and decreases in gamma band connectivity between L-DLPFC/subgenual anterior cingulate cortex (sACC), were correlated with improvements in depressive symptoms.

**Conclusions**: Our results suggest that non-invasive intervention techniques, such as rTMS, modulate the ongoing activity of depressive circuits targeted for DBS, and that MEG can capture these changes. Gamma oscillations may originate from GABA-mediated inhibition, which increases synchronization of large neuronal populations, possibly leading to increased long-range FC. We postulate that responses to rTMS could provide valuable insights into early evaluation of patient candidates for DBS surgery.

## Introduction

Major depressive disorder (MDD) is characterized by depressed mood, anhedonia, irritability, poor concentration, feelings of hopelessness, thoughts of self-harm, inappropriate guilt, and abnormal appetite and sleep patterns. About a sixth of the American population will suffer from clinical depression in their lifetime. Because of its significant societal and economic impact on the general population, both the underlying mechanism(s) of MDD and markers of individual response to treatment are important areas of ongoing research.

Previous studies have proposed that MDD is a circuit-based disorder in which several regions of the brain (prefrontal cortex, amygdala, striatum, pallidum and medial thalamus) are functionally aberrant (Drevets et al., 1992). Activity in the limbic regions, specifically the amygdala and anterior cingulate cortex (ACC; Brodmann’s area 25), is associated with depressive symptoms. Several hypotheses have been proposed to characterize MDD. The monoamine hypothesis (Krishnan and Nestler, 2008; Pittenger and Duman, 2008) suggests that a decrease in monoamine function can lead to depression. Among the monoamine projections from mid-brain to brainstem nuclei are: (1) dopamine from the ventral tegmental area (VT area); (2) serotonin from dorsal raphe in the periaqueductal gray area (PAG); and (3) noradrenaline from locus coeruleus. Secondary neuroplasticity on a cellular level is also induced by some antidepressants that acutely increase synaptic monoamines. In addition, the brain-derived neurotrophic factor (BDNF) hypothesis (Nestler and Carlezon, 2006; Krishnan and Nestler, 2008; Pittenger and Duman, 2008) implies that an increase in BDNF at the VT area and nucleus accumbens (NAc) may increase neurovegetative symptoms associated with depression.

Due to the heterogeneity of MDD, about a third of the patients suffering from the disorder are resistant to pharmacological antidepressants and/or psychotherapy. Neuromodulation therapies have emerged as potential treatments for depression. Dorsolateral prefrontal cortex (DLPFC) and ventromedial prefrontal cortex (VMPFC) are two of the main targets of neuromodulation therapy. These two targets exhibit opposite metabolic activity in the pathological depressed state (VMPFC: hyper, DLPFC: hypo; Ressler and Mayberg, 2007; Koenigs and Grafman, 2009; Kopell et al., 2011; Pathak et al., 2013). Deep brain stimulation (DBS) at the subgenual ACC (sACC) of the VMPFC network has shown promising results (Mayberg et al., 2005; Lozano et al., 2008; Mayberg, 2009; Hamani et al., 2010, 2011; Holtzheimer et al., 2012). The mechanisms of action in DBS are likely complex; however, a suppression of activity at the sACC has generally been reported to be correlated with improvement in depressive symptoms. Conversely, excitatory, noninvasive stimulation at the DLPFC via repetitive transcranial magnetic stimulation (rTMS) has efficaciously ameliorated depressive symptoms for patients (O’Reardon et al., 2007; Fitzgerald et al., 2009; Herbsman et al., 2009; Downar and Daskalakis, 2013). Lesion studies and advances in neuroimaging have provided evidence of the role of PFC in depression. In this study, we applied excitatory rTMS at the DLPFC because of its superficial location and because it plays a major role in higher cognitive functions and affective regulation.

rTMS alters neuronal activity directly beneath the excitation coil and at distant associated areas, as mediated by functional connections (Walsh and Cowey, 2000; Goodwin and Butson, 2015). Slow rTMS (<1 Hz) has been shown to be inhibitory, whereas fast rTMS (>5 Hz) is considered excitatory (Chen et al., 1997; Nakamura et al., 1997; Burt et al., 2002; Lefaucheur, 2008). Recent studies have investigated TMS as both a priming method and a treatment option for depression (Nahas et al., 2001; O’Reardon et al., 2007; Herbsman et al., 2009). The stimulation location and intensity, which are determined based on resting motor threshold (RMT), are important parameters in rTMS therapy. Lastly, magnetoencephalography (MEG) or electroencephalography (EEG) have been used to investigate local responses and remote interactions induced by rTMS (Siebner et al., 2009; Thut and Pascual-Leone, 2010).

In terms of possible biomarkers of MDD, several studies have investigated electrophysiological correlates as predictors of clinical response. Pre-treatment increase in theta power at the ACC as computed with EEG source imaging may reflect increased metabolism in ACC and is correlated with anti-depressive response (Pizzagalli, 2011; Spronk et al., 2011; Olbrich and Arns, 2013). Greater alpha power and hemispheric asymmetry have also been reported as potential predictors of response (Ulrich et al., 1984; Bruder et al., 2008; Breitenstein et al., 2014). In addition to resting-state studies, event-related potentials (ERPs) have been assessed in context of depression. Strong pre-treatment loudness dependent auditory evoked potentials (LDAEPs; Gallinat et al., 2000; Juckel et al., 2007) and P300 (Kalayam and Alexopoulos, 1999) have been associated with antidepressive response after drug treatment. These biomarkers in response to an auditory stimulus are modulated by the serotonergic system (Breitenstein et al., 2014).

We used resting-state MEG to assess possible longitudinal changes induced by high-frequency rTMS therapy. MEG source imaging is a functional neuroimaging method that senses magnetic fields generated by neural activity (Baillet et al., 2001). Specifically, scalp MEG recordings are generated essentially by synchronous postsynaptic currents driven by assemblies of pyramidal neurons oscillating over a relatively wide frequency spectrum. MEG imaging has excellent temporal resolution (millisecond) and magnetic fields are less distorted by skull and scalp tissues than EEG signals. This enables more robust modeling of neural generators in terms of mapping local activity and interregional coupling, based on time-dependent measures (Baillet et al., 2001; Gross et al., 2013).

Choosing left-dorsolateral prefrontal cortex (L-DLPFC) as a target for stimulation remains ambiguous due its broad anatomical definition. In a resting-state functional magnetic resonance imaging (fMRI) study (Fox et al., 2012a), measures from healthy participants were used to develop a connectivity-based targeting strategy to identify optimized L-DLPFC coordinates for stimulation in depressed patients. The approach included a paired comparison of the functional connectivity (FC) between two sites that previously showed clinical significance, and a correlation analysis between connectivity measures and clinical efficacy based on a model developed by Herbsman et al. (2009). The results indicate that better clinical efficacy was obtained when L-DLPFC coordinates were negatively correlated with sACC, which accounted for 70% of the variance in clinical efficacy. This is in line with the view of MDD as a “network disease” because it affects multiple, anatomically distributed brain regions.

FC is a rapidly developing subfield of neuroimaging that has contributed to better definition and testing of network models in clinical and cognitive neuroscience (Schoffelen and Gross, 2009; Sakkalis, 2011). In line with the theory of communication through coherence as a vehicle of FC in the brain, we computed magnitude-squared coherence between regions of interest (Fries, 2005). Coherence identifies significant frequency-dependent correlations between two time variant signals. It is sensitive to power and phase of neural oscillations and measures relationship stability.

We emphasize that the efficacy of rTMS therapy in treating MDD has previously been established and is not the focus of this study. Rather, the current study aims to identify objective indicators of antidepressive response to rTMS using functional neuroimaging. The long-term goal is to predict which patients will respond to anti-depressive treatment and to customize neuromodulation therapy accordingly. We are interested in two primary questions: (1) Which depressive circuits previously targeted for DBS are modulated by successful rTMS therapy for MDD? (2) Can changes in FC predict clinical response? Our hypothesis is that modulation of the L-DLPFC will be reflected through longitudinal changes in the power spectrum of local neural oscillations, along with changes in FC with other regions of the depressive circuit.

The major contribution of this study is to provide a bridge between physiological markers of MDD (neuroimaging) and clinical response to treatment. In the “Materials and Methods” Section, we describe the acquisition of high-temporal resolution MEG before, during and after delivery of high-frequency rTMS. Next, we present the approach of combining MEG source imaging with neuronavigated-rTMS to quantify how changes in depressive symptoms correlate with modulating specific circuits. In the results section, we report the significance of engaging specific functional connections in the fronto-limbic circuit for effective MDD improvement demonstrated through frequency-dependent connectivity analysis. Lastly, in the discussion section, we highlight the advances in source-imaging analysis techniques that have enabled the current study along with the limitations of those methods. We also discuss the relevance of our findings in the context of the field of neuromodulation and MDD.

## Materials and Methods

### rTMS Delivery

High frequency (10 Hz) rTMS was administered to the L-DLPFC (Brodmann areas 9 and 46) in five MDD patients (*n* = 5). Patient selection and the decision to treat using rTMS was made through standard care, independent of this study. In tandem with rTMS treatment, we enrolled patients into a research study approved by the Institutional Review Board (IRB) at the Medical College of Wisconsin. During treatment, a total of 1000 biphasic pulses were delivered in 25-s cycles (5 s on, 20 s off) over 20 trains per session using a Magstim Rapid stimulator (Magstim Company Limited, Whitland, UK). Treatment was delivered for five 15-min sessions per week over 4 weeks. Stimulation was applied at 110% of RMT, which was determined by stimulating the hand-knob of motor cortex. Periodic behavioral assessments were performed with the Montgomery-Asberg depression rating scale (MADRS).

### Anatomical Imaging

**Figure 1:**
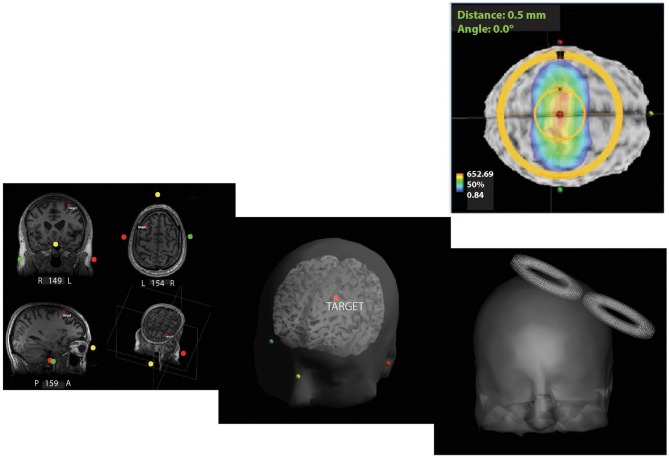
**Neuronavigation for repetitive transcranial magnetic stimulation (rTMS) therapy.** Screenshots from ANT software outline the pipeline used to target and administer neuro-navigated rTMS. Subject-specific magnetic resonance imaging (MRI) was used for 3D reconstruction of the head volume. High-frequency rTMS (10 Hz) at 110% of the resting motor threshold (RMT) was then applied at the left-dorsolateral prefrontal cortex (L-DLPFC) with real-time targeting using the ANT neuro-navigation system. (+*x* towards the nose, +*y* towards the left ear, and +*z* in the superior direction with the origin at the center of the head).

High-resolution (3T) structural magnetic resonance imaging (MRI) was acquired before treatment to assist with targeting and navigation. It was used for definition of TMS targets and registration with MEG imaging using FreeSurfer (Dale et al., 1999; Martinos Center for Biomedical Imaging) and Brainstorm (Tadel et al., 2011). A Visor neuronavigation system (ANT Neuro, Enschede, Netherlands) was used for anatomical targeting, and to record the position and orientation of the TMS coil during each pulse (Figure 1).

### Functional Imaging

MEG was recorded at 2000 Hz using a 306-channel VectorView system (Elekta Neuromag Ltd, Helsinki, Finland). EOG and EKG were also recorded simultaneous to MEG for *post hoc* artifact removal. MEG was acquired at four time points: before treatment, halfway through the treatment course (2 weeks), at the end of the treatment course (4 weeks), and 2 months after the end of treatment. Empty-room recordings were also collected at each acquisition for noise modeling in source imaging. During MEG, the subject was supine under the MEG sensor array. A total of 24 min of data was collected in four 6-min runs. Each condition (eyes-open and eyes-closed) was repeated once.

### Source Analysis

MEG data for the eyes-closed condition were analyzed for cortical source modeling, power spectral density (PSD), and FC using Brainstorm, with default imaging parameters (Tadel et al., 2011). The results were assessed to identify longitudinal changes in PSD over standard frequency bands (delta = 2–4 Hz; theta = 5–7 Hz; alpha = 8–12 Hz; beta = 15–29 Hz; gamma = 30–59 Hz) of ongoing oscillatory neural activity. Continuous signals were cleaned from physiological and other artifacts with the signal-space separation (SSS) method (Taulu et al., 2005) and further corrected with specific signal-space projectors (SSP), a method based on principal components analysis to eliminate artifacts caused by eye-blinks and heartbeats; all were performed using default settings in Brainstorm. The data was then processed with a 60-Hz notch filter and a 2-Hz high-pass filter to remove residual noise due to the power line and slow drifts of sensor DC offset.

The weighted minimum-norm estimate (wMNE), a subtype of distributed source models, was used to generate the source image maps, with default imaging parameters in Brainstorm. Elementary dipole current sources were constrained perpendicularly to the individual cortex; additive sensor noise was modeled with a Gaussian distribution with known covariance statistics estimated from empty-room recordings. The Welch periodogram was used to obtain the PSD of each individual source time series on the cortex. Signals from the L-DLPFC were averaged over the stimulation area to determine the power at that region (Goodwin and Butson, 2015). For this analysis, L-DLPFC was defined based on the Desikan-Killiany atlas (Desikan et al., 2006) from FreeSurfer. A linear regression analysis was performed in MATLAB (Mathworks Inc., Natick, MA, USA) to determine if power within a particular frequency band was correlated with clinical improvement.

### Functional Connectivity

Coherence measures computed on 10-s epochs were used to determine FC between L-DLPFC and several regions of interest (ROIs) previously investigated for DBS therapy: pregenual anterior cingulate cortex (pACC) and sACC, NAc, and amygdala. The Desikan-Killiany atlas (Desikan et al., 2006) from FreeSurfer was used to define the ROIs. In addition, a cluster analysis was conducted in MATLAB and visualized in SCIRun (Center for Integrative Biomedical Computing, University of Utah). For this analysis, each node on the L-DLPFC surface was assigned a mean connectivity score based on the average of its coherence with signals at each of the nodes in the other ROIs (pACC, sACC or NAc). For example, if the L-DLPFC was parcellated into N1 nodes and sACC was parcellated into N2 nodes, a coherence matrix of N1 × N2 would be computed to reflect the connectivity between L-DLPFC and sACC. Coherence would then be averaged for all nodes in the sACC to assign one connectivity score to each node of the L-DLPFC, collapsing the dimensions of the initial matrix to N1 × 1. Mean connectivity was represented with a colormap to visualize the differential effects of sub-regions in the L-DLPFC.

### Statistical Analysis

We used a multiple regression model (Equation 1) to investigate the relationship between change in behavior (MADRS) and change in brain activity, controlling for time and subject effects which were treated as categorical variables. We performed this regression analysis for each frequency band in the source analysis (L-DLPFC) and the connectivity analysis (Amygdala, sACC, pACC and NAc). We did not correct for multiple comparisons given the exploratory nature of this study and its small sample size. All tests were two-tailed and statistical significance was assessed at the 0.05 level.

(1) ΔMADRS~Subject+Time+ΔBrainActivity

## Results

**Figure 2:**
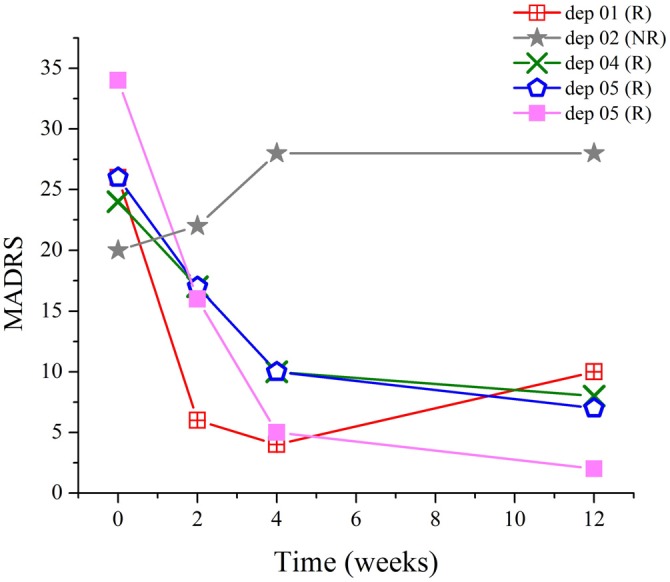
**Relationship between Montgomery-Asberg depression rating scale (MADRS) scores and time.** Drastic improvement occurred in responders during the course of treatment, which persisted at least for another 8 weeks, as demonstrated by the scores at follow-up.

Data from five MDD patients were analyzed in this study. Of them, four were responders and one was a non-responder using the clinical response criterion of a 40% reduction in MADRS scores from baseline. Stimulation was applied to the L-DLPFC using a predefined target coordinate on an anatomical MRI. By midpoint, an improvement in depressive symptoms was observed in responders (Table 1). These participants continued to improve during treatment, and were still in remission at the 2-month follow-up (Figure 2).

**Table 1:** **Summary of Montgomery-Asberg depression rating scale (MADRS) scores for all subjects over 4 weeks of repetitive transcranial magnetic stimulation (rTMS) therapy and at 12-week follow-up (R, Responder; NR, Non-responder)**.

MADRS scores over the course of rTMS for depression					
	Dep01 (R)	Dep02 (NR)	Dep04 (R)	Dep05 (R)	Dep06 (R)
Baseline	26	20	24	26	34
Midpoint	6	22	17	17	16
Endpoint	4	28	10	10	5
Follow-up	10	28	8	7	2

### Increase in High-Frequency Power at the L-DLPFC Correlates with an Improvement in Depressive Symptoms

**Figure 3:**
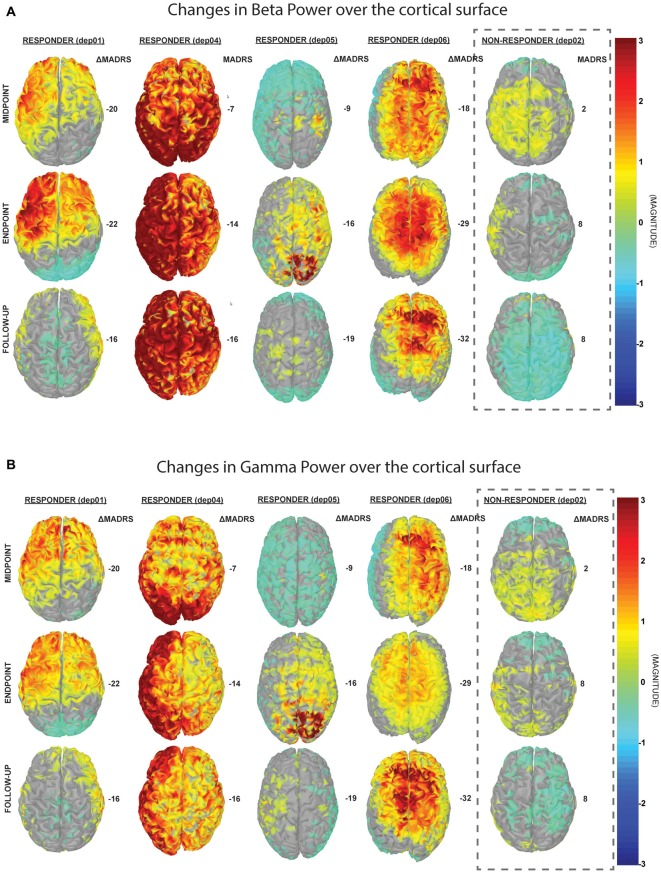
**Changes in cortical power spectral density (PSD).** These cortical maps display the normalized difference in PSD from baseline in **(A)** beta (13–29 Hz) and **(B)** gamma (30–59 Hz) frequency bands. For the responders, there is a trend towards increase in high-frequency power bilaterally, which corresponds to improvements in depressive symptoms, as determined by the change in MADRS (right column for each patient).

**Figure 4:**
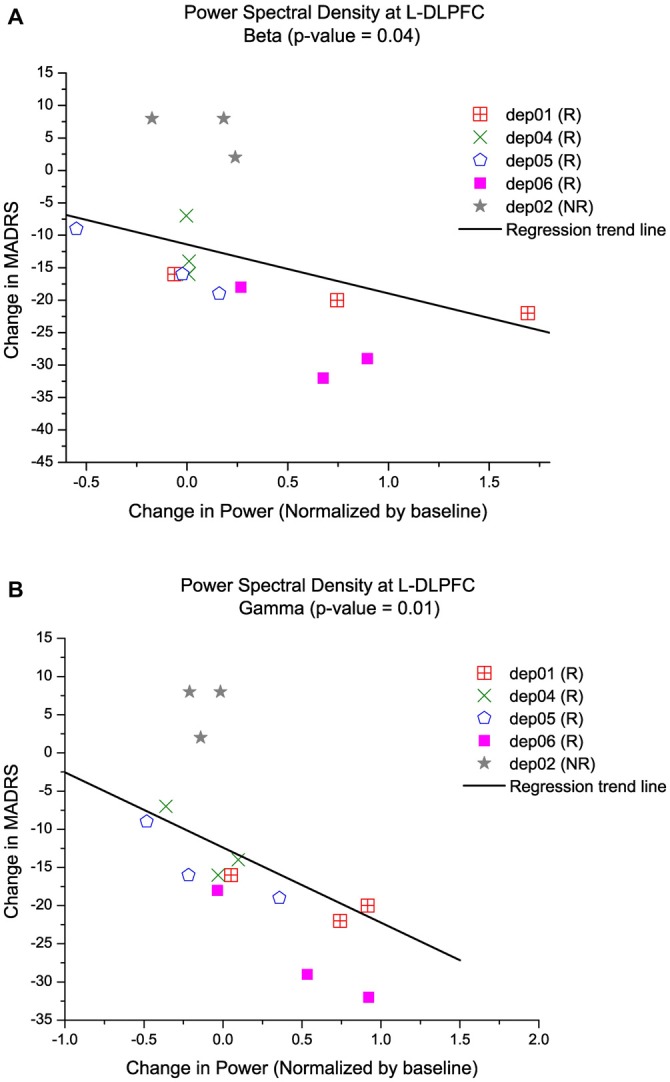
**Increase in high-frequency power at the L-DLPFC is correlated with an improvement in depressive symptoms.** Multiple regression models of MADRS vs. PSD controlled for time and subject, show that an increase in high-frequency power is indicative of lower MADRS or better clinical outcome. Each symbol represents a different subject and the trend line is a reflection of the model to display the relationship between **(A)** MADRS and beta power, and **(B)** MADRS and gamma power.

The results from the PSD analysis indicated that an increase in ongoing gamma (γ; 30–59 Hz) power (*p*(γ) = 0.01) and beta (12–29 Hz; β) power (*p*(β) = 0.04) at the L-DLPFC was significantly correlated with an improvement in depressive symptoms (Table 2). A power distribution over cortical maps (Figure 3) showed a marked increase of high-frequency power bilaterally in the frontal regions of responders throughout the course of treatment. These observations were corroborated by a multiple regression analysis (Figure 4).

**Table 2:** **Summary of statistics from multiple regression models**.

	Delta		Theta		Alpha		Beta		Gamma	
	Coeff	(*p*-val)	Coeff	(*p*-val)	Coeff	(*p*-val)	Coeff	(*p*-val)	Coeff	(*p*-val)
L-DLPFC (PSD)	−3.27 ± 2.30	0.148	−5.66 ± 2.96	0.06	−8.59 ± 4.70	0.07	−7.56 ± 3.55	0.04*	−9.84 ± 3.42	0.011*
Amygdala (FC)	−10.24 ± 4.80	0.040*	−0.53 ± 3.53	0.86	4.78 ± 4.99	0.29	2.16 ± 2.97	0.42	−3.71 ± 5.14	0.420
NAc (FC)	−3.12 ± 3.49	0.320	−3.91 ± 3.43	0.22	0.19 ± 7.84	0.98	−0.12 ± 6.73	0.98	−5.39 ± 6.68	0.370
sACC (FC)	−0.70 ± 1.82	0.660	−0.77 ± 1.91	0.65	2.25 ± 5.92	0.67	4.79 ± 2.94	0.09	2.48 ± 6.25	0.650
pACC (FC)	−0.78 ± 1.01	0.390	−0.76 ± 1.07	0.43	−3.03 ± 4.91	0.49	3.96 ± 6.63	0.50	0.11 ± 3.58	0.970

PSD, power spectral density; FC, functional connectivity; *Indicates significance at α = 0.05.

### Opposite Effects of Functional Connectivity in High and Low Frequency Bands Between L-DLPFC and Remote Regions

**Figure 5:**
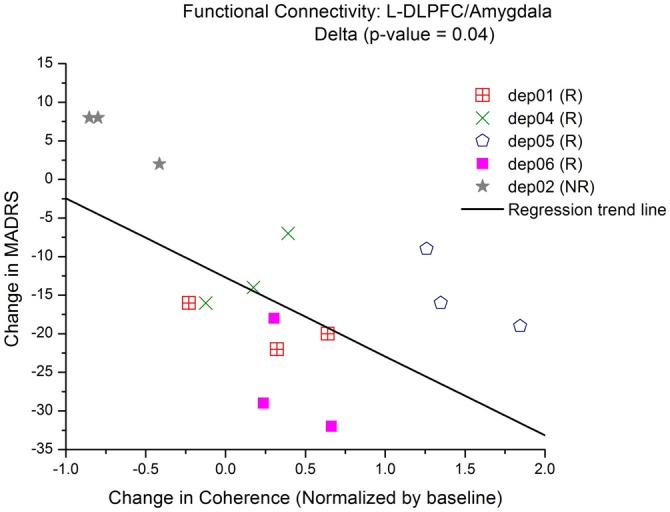
**Increase in low-frequency connectivity between L-DLPFC and amygdala.** Multiple regression models of MADRS vs. coherence show that there is a significant linear relationship between connectivity and an improvement in depressive symptoms. Each symbol represents a different subject and the trend line is a reflection of the model to display the relationship between MADRS and coherence in the delta band.

**Figure 6:**
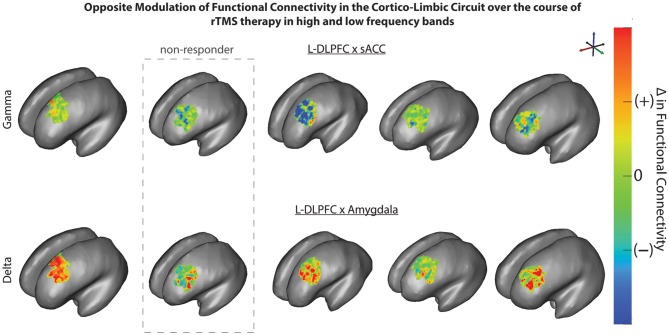
**rTMS modulates connectivity over the L-DLPFC in responders.** L-DLPFC/amygdala coherence in delta band (bottom) increased from baseline to endpoint over the entire area of stimulation for responders. An opposite effect was observed with a decrease in L-DLPFC/subgenual anterior cingulate cortex (sACC) coherence in gamma band (top) from baseline to endpoint over the entire area of stimulation for responders. The nodes in L-DLPFC contributing to change in connectivity are common for both amygdala and sACC for all patients.

Changes in FC of L-DLPFC and several ROI in the depressive circuit were assessed. An increase in mean coherence in the delta band (2–4 Hz) correlated with a better clinical outcome for depression in the connectivity analysis between L-DLPFC and amygdala (*p*(δ) = 0.04; Table 2), as demonstrated by a multiple regression model of MADRS with coherence (Figure 5). A qualitative cluster analysis also demonstrated an increase in DLPFC-amygdala coherence from baseline to endpoint in delta band over the entire area of stimulation for the responders when mapped onto the cortex. In contrast, a decrease in DLPFC-sACC coherence in the gamma band was observed. Furthermore, the nodes involved in modulating connectivity were common for amygdala and sACC (Figure 6).

## Discussion

The target of rTMS stimulation was the L-DLPFC as this region tends to show diminished function in individuals with MDD. This area is implicated in working-memory, attention, and long-term memory storage and consolidation and previous studies have shown improvements following stimulation in frontal cortices (Curtis and D’Esposito, 2003; Fregni et al., 2005). We did not formally test working memory, but questions relating to recall, attention and orientation were asked daily to ensure there were no negative consequences of stimulation. Our results support the hypothesis that longitudinal changes from rTMS therapy are reflected in the PSD and connectivity analyses of ongoing neural oscillations in the resting-state. We emphasize that the cohort for this study consisted of treatment-resistant patients who failed previously at least two treatment strategies; this implies that a placebo response is unlikely and cannot fully explain the improvement in MDD symptoms. Furthermore, we show that correlation of such improvement with the neurophysiological changes captured by MEG is an objective way of assessing rTMS therapy.

A local increase in beta and gamma power at the L-DLPFC was correlated with an improvement in depressive symptoms. A comparison of PSD in the right occipital cortex as a control region did not result in a trend similar to the one observed at the L-DLPFC. We posit that this increased power may reflect a rise in metabolic activity of the DLPFC, which was shown to contribute to improvement of symptoms. High-frequency oscillations in the beta and gamma range have previously been linked to increased attentional processes (Benchenane et al., 2011). One mechanism of gamma modulation at a pharmacological level involves GABAergic interneurons. These cells contribute to synchronization of the pyramidal neurons in the DLPFC during tasks that require increased cognitive effort. Furthermore, GABA-mediated inhibition was shown to be sufficient in generating gamma oscillations (Barr et al., 2009).

Previous evidence also points to GABAergic involvement in antidepressant response (Bajbouj et al., 2005; Yue et al., 2009). Animal models of depression have shown that GABA agonists have antidepressant activity (Petty, 1995; Yue et al., 2009). If inhibition is facilitated by fast acting GABA_A_ receptors, oscillation frequency will correspond to the gamma band (Buzsaki, 2006). Additionally, increased cortical beta is known to enhance the effect of GABA. Consequently, it is plausible that increased cortical beta activity, which was also observed in this study, boosted the effect of GABA leading to inhibition that increased gamma power. Another consequence of GABA-mediated inhibition is synchronization of large neuronal populations, leading to increased long-range FC mediated by interregional coherence (Fingelkurts et al., 2004). Alternatively, post-synaptic targets are also known to up-regulate expression patterns of GABAergic receptors or change their expression pattern of the GABAergic receptor subtypes that could lead to changes in behavioral outcome (Lloyd et al., 1989; Petty, 1995; Brambilla et al., 2003). Though GABA may be involved, it is difficult to determine exactly how it is exerting its effects in this case.

Changes in synaptic plasticity are directly related to changes measured in FC and the strength of a synapse can further dictate the effect of inhibition (Buzsaki, 2006). The depressive circuit targeted for DBS includes amygdala, pACC, sACC, and the NAc (Ressler and Mayberg, 2007; Krishnan and Nestler, 2008; Holtzheimer and Mayberg, 2011). Recent developments in source imaging methods enable a more accurate representation of neural activity at deeper sources. Attal and Schwartz (2013) demonstrated that realistic anatomical and electrophysiological models of deep brain activity (DBA) allow for the estimation of contributions from deep sources such as thalamus and amygdala at MEG sensors. In the present study, the measures extracted are based on integration over a large amount of time samples (power spectrum density and coherence). Therefore, these are equivalent to a form of averaging effect (enhancement of SNR), which indicates that signal detection was substantially improved. Additionally, source imaging was obtained using the MRI obtained from each participant to account for individual anatomy and noise statistics were captured using an empty-room recording, and embedded into the source estimator. The most effective targets for DBS are sACC and amygdala. This evidence further lends credence to our results that indicate the significance of modulating connectivity between these specific regions.

Activity in sACC contributes substantially to limbic-cortical dysregulation (Mayberg, 1997; Greicius et al., 2007). Depression severity has been shown to correlate positively with FC in sACC. In addition, increased connectivity of the default mode network (DMN) in depression was also driven by this region (Greicius et al., 2007). Fox et al. (2012b) also showed that the magnitude of relative disconnectivity (anticorrelation of fMRI signals) between L-DLPFC and ACC was indicative of depression severity. The distinct role of subregion FC is corroborated by differential anatomical connectivity of the pACC and sACC, suggesting a strong connection of pACC to frontal regions vs. sACC to limbic regions (Johansen-Berg et al., 2008). These studies imply that when an individual is improving, anti-correlation between sACC and DLPFC weakens, which would in turn manifest in a coherence decrease, supporting our results for the cluster analysis that clearly show the nodes involved in decreased gamma coherence between L-DLPFC and sACC (Figure 6). This further suggests that part of the antidepressant mechanism enabled by rTMS may be in the remote suppression of the sACC, which has been shown to be hypermetabolic in depressed individuals.

Another finding is that an increase in coherence was observed in the delta band for amygdala (Figures 5, 6). Different frequency bands may enable the multiplexing of channels of communication for different functional brain networks (Lakatos et al., 2005). Communication through coherence is thought to be regulated by inhibitory windows (Benchenane et al., 2011). FC in lower frequency bands has been associated with higher cognitive tasks. Previous research has also demonstrated that anatomically distributed EEG coherence increased in lower frequencies, particularly the delta band during creative thought (Petsche, 1996; Gruzelier, 2009). Furthermore, neuroimaging and neurophysiological studies have reported that long-distance integration is coordinated by lower frequencies, whereas short-distance networks are coordinated by higher frequencies (Buzsaki, 2006; Leuchter et al., 2012). In particular for mood disorders, an fMRI study (Anand et al., 2005) showed an increase in low-frequency blood-oxygen-level dependent (BOLD) fluctuation (LFBF) correlation between ACC and limbic regions in patients after treatment for depression. Another study (Chepenik et al., 2010) identified a negative correlation in low-frequency resting-state fMRI activity between the ventral PFC and amygdala in healthy subjects compared to those with bipolar disorder. These reports corroborate our current findings of increased connectivity between L-DLPFC and amygdala in the delta band correlating with better clinical outcome.

According to an MEG study of resting-state FC (Hillebrand et al., 2012), there is a positive correlation between patterns of source power and FC. A modeling study (Chawla et al., 1999, 2000) also showed increased phase locking between two connected neuronal populations resulting in increases in both power and FC. Though we did not note a statistical significance in the connectivity analysis between L-DLPFC and sACC in our multiple regression analysis, we would allude to the results of our cluster analysis that align with these previous findings and demonstrate decreased gamma connectivity between the two regions.

Lastly, the modulation in coherence between frontal and limbic regions was observed over the entire area of stimulation for responders, when mean connectivity was mapped onto the L-DLPFC. This finding implies that stimulation of responders engaged functional connections that were relevant for clinical improvement and nonresponders were effectively lacking in engaging those essential connections. In a previous study investigating chronic epidural cortical stimulation (EpCS) for depression (Kopell et al., 2011; Pathak et al., 2013), stimulation using the mixed mode polarity, in which the configuration of the active electrode alternated between anode and cathode in a pseudorandom manner, was effective in achieving an antidepressant response. This response was likely due to recruitment of a wider range of neurons owing to characteristic differences in anodic and cathodic stimulations (Wongsarnpigoon and Grill, 2008; Pathak et al., 2013). Another study (Riva-Posse et al., 2014) investigating fiber tracts concluded that the involvement of bilateral forceps minor, the cingulate bundle, and short descending midline fibers was predictive of clinical response. Riva-Posse et al. (2014) emphasized that the anatomical location of active electrodes was not the source of variability, but instead it was the hub location where these three fibers intersected that determined potential of response.

In terms of study design, a sham group was not included because the study was not testing actual treatment efficacy of TMS. Instead, our goal was to conduct a longitudinal study to evaluate neuroimaging changes in patient volunteers showing MDD clinical improvements following TMS treatment. While this still does not entirely rule out a possible placebo effect, it remains highly unlikely since these patients had failed at least two previous treatment options that are otherwise effective. Further, the STAR * D report elegantly explains that there is a reduction in remission rates as the number of treatment steps increase (Rush et al., 2006).

When working with resting state signals, we cannot disregard the possibility of negative self-talk or rumination. Future studies should attempt to control for this. However, this is a pilot study. Further, resting state here only implies task-free; how an individual controls their thoughts are beyond the scope of this study. In fact, this state of rumination might reflect the resting state of an MDD patient. Additionally, our data has been averaged over several segments of time for the analysis. If these occurrences are spontaneous, it is likely that their effect will be eliminated.

There are a few limitations that should be addressed in future studies as the field advances. First, interaction of TMS with medication is not clearly understood. There is a possibility that changes in medication can affect cortical excitability which might necessitate a re-evaluation of the TMS “dose”. Secondly, we analyzed data from a relatively small sample size. As this is a small patient cohort, we did not correct for multiple comparisons which could increase the probability of a Type I error. However, the longitudinal nature of our study design provides the opportunity to observe changes at an individual level over the course of neuromodulation therapy. With a push towards precision medicine, it becomes important to measure improvement in a patient-specific manner. Additionally, our findings regarding neuroimaging correlates of improvement in depression take into account response and non-response, and the results are selective for specific anatomical regions.

## Conclusion

The primary finding from this study is that in addition to local changes, high-frequency rTMS modulates remote nodes of the depressive circuit during an effective clinical response. This conclusion was reached by combining anatomical targeting (from rTMS navigation) with source analysis and FC as determined from MEG. We postulate that this approach could fill two important gaps in our current knowledge. First, it could improve identification of specific regions and functional connections to target in order to maximize the therapeutic response. Second, this approach could provide insights about common mechanisms for different types of neuromodulation therapy including rTMS, cortical stimulation and DBS.

## Author Contributions

YP, OS, SB, ZL, and CRB participated in manuscript preparation and editing; YP and CRB conducted the analysis to determine the effect of repetitive transcranial magnetic stimulation on changes in the power spectral density and functional connectivity; OS was the psychiatrist for the clinical study and assisted with the administration of therapy; ZL assisted with the acquisition of neuroimaging data; SB guided the analysis and interpretation of the neuroimaging data.

## Conflict of Interest Statement

The authors declare that the research was conducted in the absence of any commercial or financial relationships that could be construed as a potential conflict of interest. The reviewer CP and the handling Editor declared their shared affiliation, and the handling Editor states that the process nevertheless met the standards of a fair and objective review.
